# High Drug Resistance in Feline Mammary Carcinoma Cell Line (FMCm) and Comparison with Human Breast Cancer Cell Line (MCF-7)

**DOI:** 10.3390/ani11082321

**Published:** 2021-08-06

**Authors:** Ana Salomé Correia, Rita Matos, Fátima Gärtner, Irina Amorim, Nuno Vale

**Affiliations:** 1 OncoPharma Research Group, Center for Health Technology and Services Research (CINTESIS), Rua Dr. Plácido da Costa, 4200-450 Porto, Portugal; anncorr07@gmail.com; 2 Department of Molecular Pathology and Immunology, Institute of Biomedical Sciences Abel Salazar (ICBAS), University of Porto, Rua de Jorge Viterbo Ferreira 228, 4050-313 Porto, Portugal; ritam@ipatimup.pt (R.M.); iamorim@ipatimup.pt (I.A.); 3 Institute of Molecular Pathology and Immunology of the University of Porto (IPATIMUP), Rua Júlio Amaral de Carvalho 45, 4200-135 Porto, Portugal; fgartner@ipatimup.pt; 4 i3S, Instituto de Investigação e Inovação em Saúde da Universidade do Porto, Rua Alfredo Allen 208, 4200-135 Porto, Portugal; 5 Department of Community Medicine, Health Information and Decision (MEDCIDS), Faculty of Medicine, University of Porto, Al. Prof. Hernâni Monteiro, 4200-319 Porto, Portugal

**Keywords:** FMCm cells, MCF-7 cells, drug combination, drug resistance

## Abstract

**Simple Summary:**

The repurposing and combination of drugs are important therapeutic strategies in cancer therapy, being important for combating drug resistance and improving therapeutical regimens. This paper outlines the use of candidate drugs that are to be repurposed, and combines clinically approved drugs in breast cancer, aiming to understand the response of feline mammary carcinoma cells to this therapeutic approach, previously applied in human breast cancer cells. By using cell viability assays, we revealed that feline mammary carcinoma cells were highly resistant to these tested approaches, contrasting with human breast cancer cells.

**Abstract:**

Drug repurposing and drug combination are important therapeutic approaches in cancer therapy. Drug repurposing aims to give new indications to drugs, rather than the original indication, whereas drug combination presupposes that the effect that is obtained should be more beneficial than the effect obtained by the individual drugs. Previously, drug repurposing and the combination of different drugs was evaluated in our research group against human breast cancer cells (MCF-7 cells). Our results demonstrated that the response obtained through the combination of drugs, when compared with the single drugs, led to more synergic responses. Therefore, using potential drugs for repurposing, combined with a reference drug in breast cancer (5-Fluorouracil), was the major aim of this project, but for the first time using the feline mammary carcinoma cell line, FMCm. Surprisingly, the feline neoplastic cells demonstrated considerable resistance to the drugs tested in isolation, and the combination was not effective, which contrasted with the obtained MCF-7 cells’ response.

## 1. Introduction

In female cats, mammary cancer is the third most common neoplasia. 85–90% of these pathologies present a malignant phenotype, having extremely high mortality rates and a mean survival time of about one year [1,2]. Besides local recurrence, metastatic disease is also commonly observed, mainly involving regional lymph nodes, lungs, pleura, and liver [3]. The most common therapeutic approach is surgery, which can be used alone or, depending on the status of the disease, in combination with chemotherapeutic agents. Combination chemotherapy using doxorubicin and cyclophosphamide, or carboplatin is the more common chemotherapeutic regimen [4]. Additionally, emerging studies suggest that histone deacetylases inhibitors and microtubules inhibitors are potential candidates for the treatment of feline mammary carcinoma, inducing cell death [5]. Previously, the FMCm cell line (Feline Mammary Carcinoma) was demonstrated to be highly tumorigenic and metastatic, leading to the observation of both primary and metastatic lesions, particularly in animals that have the expression of all the complex of cadherin-catenin [6]. Recently, our research group reported a new model of the drug combination, using classical and repurposed drugs. This combination model proved to be very effective, because the repurposed drug displayed a better profile than the drug currently used in therapy, and the effect of both generated even more satisfactory results when tested in the MCF-7 cell line [7]. Drug repurposing is an approach that aims to give new indications for already approved drugs. This methodology has the advantage of allowing lower inherent costs and shorter periods of time until the final approval of the drug, because details about side effects and other parameters such as pharmacokinetics and interactions between drugs are already investigated [8]. The investigation of drugs approved for indications that do not include cancer can most likely offer beneficial treatment options for oncologic patients [9]. Some examples are presented in Table 1. Regarding drug combination, studies point out that this approach has several benefits, such as a better overall efficacy, decreased toxicity levels and the possibility to circumvent drug resistance [10]. Thus, drug combination is increasingly used in clinical practice, highlighting oncological therapy [11]. In fact, when combined, two drugs can produce different therapeutic effects, being pharmacodynamically synergistic (when the effect of the combination is greater than the individual drugs), additive (the effect of the combination is equal to the single drugs), or antagonistic (the effect of the combination is lesser than the single drugs) [12]. Thus, if two drugs are synergic, it is possible to minimize adverse effects and improve overall efficacy. In the context of cancer, a lot of clinical trials are testing combinations that include several therapeutic modalities such as chemotherapeutic drugs, hormonal therapies, and molecularly targeted therapies [13]. However, despite a growing number of studies, the selection of treatment regimens for breast cancer remains a complex procedure. We therefore intended to study the degree of resistance of FMCm cells to most of the drugs previously used in our MCF-7 model (Table 1).

**Figure 1:**
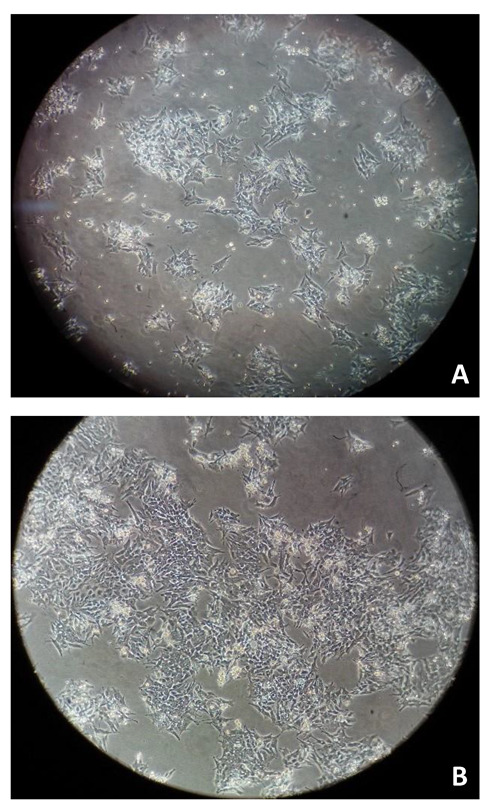
FMCm cells (**A**) with a confluency of 70%, 48 h, and (**B**) with a confluency of 80%, 72 h. All images were obtained through the optical microscope Lionheart™ FX Automated (20×).

The major aim of this project was to investigate, in FMCm cells, the response to potentially repurposed drugs combined with a clinically used drug (5-Fluorouracil) for mammary cancer therapy. The big goal is that the combination of both drugs presents beneficial effects when compared to the isolated effect of each drug. Additionally, we aimed to discern the effects triggered in these two breast cancer cell lines: feline and human. The experimental work that is described was carried out in FMCm cells (Figure 1). This cell line is characterized as a feline mammary adenocarcinoma cell line, originating from a regional lymph node metastatic lesion of a 12-years-old Japanese female cat diagnosed with a stage III primary mammary adenocarcinoma with great metastatic potential [24], being adherent, anchorage-dependent, and growing as a monolayer (Figure 1).

## 2. Materials and Methods

### 2.1. Materials

Dulbecco’s Modified Eagle’s Medium (DMEM), RPMI 1640 Medium, DMEM/F-12, Fetal Bovine Serum (FBS), and Penicillin-Streptomycin mixture were obtained from Milipore Sigma (Merck KGaA, Darmstadt, Germany). Human Insulin was purchased from NovoNordisk (Bagsværd, Denmark); Thiazolyl Blue Tetrazolium Bromide (MTT; cat. no. M5655), 5-Fluorouracil (5-FU; cat.no F6627), Verapamil hydrochloride (cat. no. V4629), 2-Acetoxybenzoic acid (Aspirin; cat. no. A2093), Losartan potassium (cat. no. 61188), Chloroquine diphosphate salt (cat. no. C6628), Cimetidine (cat. no. C4522), Itraconazole (cat. no. I6657), 9-Amino-1,2,3,4-tetrahydroacridine hydrochloride hydrate (Tacrine hydrochloride; cat. no. A79922) and Isonicotinic acid hydrazide (Isoniazid; cat. no. I3377) were purchased from Sigma-Aldrich (Merck KGaA, Darmstadt, Germany). Pravastatin (cat. no. 0010342) was purchased from Cayman Chemical Company (Ann Arbor, MI, USA).

### 2.2. Cell Culture and Treatments

Regarding the cell culture, FMCm (Passage 2; IPATIMUP, Porto, Portugal), MCF-7 (Passage 5; American Type Culture Collection, Manassas, VA, USA) and MCF-10A (Passage 7; American Type Culture Collection) were cultivated in RPMI 1640, DMEM and DMEM/F-12, respectively (10% FBS, 1% of a solution of penicillin/streptomycin (1000 U/mL; 10 mg/mL)). Additionally, DMEM/F-12 was supplemented with 2 µg/mL of human insulin, 20 ng/mL of epidermal growth factor and 1 µM hydrocortisone, as previously described [7]. All cells were incubated at 37 °C (with 5% CO_2_). Experiments were executed with cells in a 70–80% confluence. Before each experiment, cells were trypsinized (0.25% trypsin-EDTA), centrifuged (1100 rpm, 5 min), and seeded at an optimal density of 3 × 10^4^ cells/mL in 96-well plates, attaching for 24 h. For the treatment of MCF-7 and MCF-10A, all the drugs were applied in a concentration of 50 μM, as previously described [7]. For FMCm cells, all the compounds under study were dissolved in Dimethylsulfoxide (DMSO) and applied to the cells at final concentrations of 50 µM (dissolved in culture media). Controls were composed of DMSO 0.1% *v/v*.

### 2.3. Cell Viability Assays

The values of cellular viability after 72 h of treatment were determined by performing an MTT (3-(4,5-dimethylthiazol-2-yl) -2,5-diphenyltetrazolium bromide) tetrazolium reduction assay. To evaluate the viability of the cells after drug exposure, an MTT assay was performed. Cells were seeded in 96-well plates (37 °C for 24 h). Then, the treatments were added to the cells for a total time of 72 h. After that, the cell medium was removed and 100 μL of MTT solution (0.5 mg/mL in PBS) was added to each plate well. Then, the cells were incubated at 37 °C for 3 h, protected from light. Finally, 100 μL/well of DMSO was added after MTT solution removal. Absorbance readings (570 nm) were then carried out (Sinergy HT, BioTek Instruments, Winooski, VT, USA).

### 2.4. Statistical Analysis

The results are presented as the mean ± standard error of the mean (SEM). Differences between controls and cells subjected to treatment were analyzed by using a one-way ANOVA and by Dunnett’s test. Differences between the combination of drugs and the more efficacious drug of the combination were analyzed by a Student’s t-test. Statistical significance was considered when *p* < 0.05. All the tests were performed with SigmaPlot 12.0 (San Jose, CA, USA), except the Student’s t-test, which was performed with GraphPad Prism 7 (San Diego, CA, USA).

## 3. Results and Discussion

**Figure 2:**
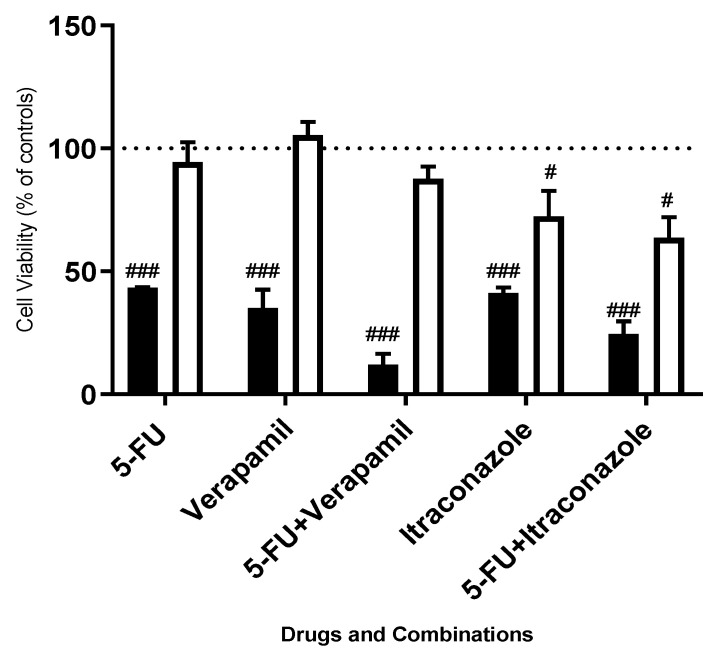
Cell viability values of 5-FU, verapamil, itraconazole and the combination of 5-FU with verapamil and itraconazole in MCF-7 cells (black bars) and MCF-10A cells (white bars), 72 h. All the compounds were added to cells in sextuplicates. The results (mean ± SEM) are presented as a percentage of the control (100%) of four independent experiments. ### *p* < 0.001 and # *p* < 0.05 vs. controls; Adapted from [7].

**Figure 3:**
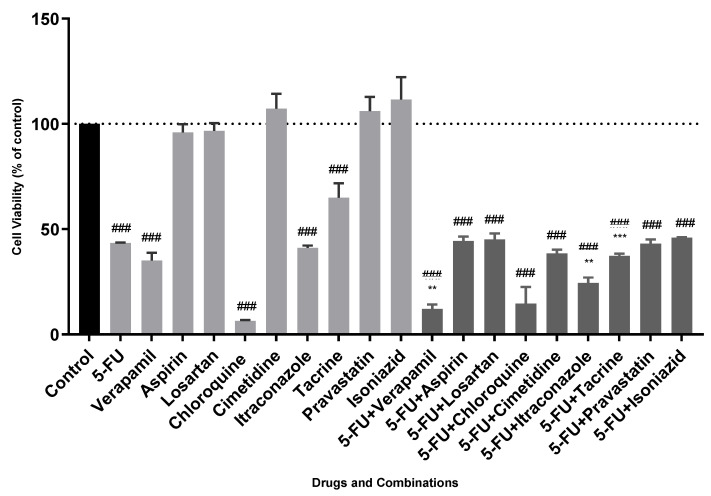
Cell viability values of single drugs and drug combinations in MCF-7 cells. All the compounds were added to cells in sextuplicates. The results (mean ± SEM) are presented as a percentage of the control (100%) of four independent experiments. ### *p* < 0.001 vs. control; ** *p* < 0.01 and *** *p* < 0.001 vs. the more effective drug of the combination. Reproduced from [7].

**Figure 4:**
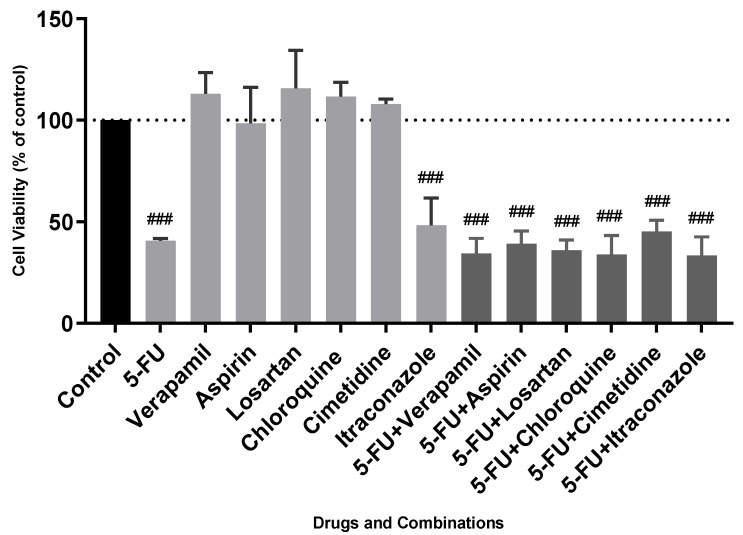
Cell viability values of single drugs and drug combinations in FMCm cells. All the compounds were added to cells in sextuplicates. The results (mean ± SEM) are presented as a percentage of the control (100%) of four independent experiments. ### *p* < 0.001 vs. control.

5-FU was chosen as the reference anti-neoplastic drug for this project, highlighting a strong profile for this drug to be used in combination therapeutic regimens in breast cancer therapy, having the potential to increase its efficacy and safety profile. Briefly, this drug, as well as its metabolites, leads to damage in RNA and DNA [25]. Based on literature research about drugs with the potential to be repurposed, as well as the interests of the research group, six and nine drugs were tested in FMCm and MCF-7 cell lines in combination with 5-FU, respectively, to make a screening of drugs to be combined with 5-FU. Each drug was used in a concentration of 50 μM, acting for 72 h. The use of a breast cell line (MCF-10A, nontumoral) compared to a breast cancer cell line (MCF-7) led us to understand that the use of the concentration of 50 μM allowed us to distinguish responses between tumoral and nontumoral cells, even with the more effective drugs (Figure 2) [7]. Thus, this is an effective concentration for cancer cells, with very few effects in normal breast cells, justifying the use of this concentration for further work. The results were obtained by a MTT viability assay (Figure 3 and Figure 4).

In MCF-7 cells, our results revealed that the drug with more efficacy regarding cell viability reduction was chloroquine (6.5 ± 0.4% of cellular viability), being more effective than single and combined drugs. The combinations of 5-FU with losartan, aspirin, cimetidine, pravastatin, tacrine, and isoniazid were not effective. However, two combinations of drugs were effective: 5-FU in combination with verapamil and itraconazole, a potential synergistic drug combination. The exposure of MCF-7 cells to 5-FU combined with these drugs led to a reduction in cellular viability (versus the more effective drug of the precise combination) of 23% for verapamil and 17% for itraconazole, respectively. Values of cell viability of approximately 12% and 25% were, indeed, obtained with the combinations of 5-FU/verapamil and 5-FU/itraconazole, respectively. Determined in our group, the obtained IC_50_ values for the more effective isolated drugs (5-FU, verapamil, itraconazole and chloroquine) are confirmed by the values presented in Table 2. The other drugs presented an IC_50_ value of more than 100 μM, highlighting the fact that they were not effective in these experimental conditions.

Regarding FMCm cells, it is possible to realize that none of the tested drug combinations efficiently reduced cell viability when compared with the single drugs. The lower cell viability value reached with drug combinations was obtained with 5-FU combined with chloroquine (33.9 ± 5.4%), which was not shown to be advantageous relative to the most effective drug of that combination (5-FU) with values of 40.6 ± 1.1%. Clearly, the drug combinations produce an effect almost equal to that of 5-FU alone, showing that the effects of the combination on cell viability are only due to the action of 5-FU and not from the combination of both drugs. In general, feline mammary carcinomas have high mortality rates, being associated with a poor prognosis. Studies in nude mice, with the FMCm cell line, demonstrated that these cells were very aggressive and had a high metastatic and tumorigenic potential [1,3,26]. An interesting finding is that, in these studies, metastasis expressed P- and E-cadherin [4] (Table 3), potentially linked with the resistance of the cells observed in our project. Indeed, the overexpression of P-cadherin in tumors that express E-cadherin is correlated with more aggressive behaviors and a worse tumor prognosis in comparison with carcinomas that express only one type of these molecules [6,27,28]. It is important to consider that the mechanism of action of the tested drugs is unknown in the oncology field. Thus, they can interact differently in FMCm cells from how they interact in MCF-7 cells, which has indeed been demonstrated in this project. For example, in the MCF-7 cells, the drug combinations 5-FU+Verapimil and 5-FU+Itraconazole appeared to show synergism, which was not observed in FMCm cells. In addition to what was mentioned above, verapamil and itraconazole may act in specific pathways when combined with 5-FU in human cells. Knowing that verapamil and itraconazole act, respectively, in inhibiting calcium channels and inhibiting the 14α-demethylase enzyme, it is expected that these factors are more related to human breast cancer and not to feline breast cancer. Another interesting example that can be observed in this study is the fact that chloroquine is effective in human breast cancer, in contrast to feline breast cancer. These data may reveal that the molecular pathways by which chloroquine acts in breast cancer are overexpressed in human cells and not in feline cells. For example, it is known that chloroquine, in cancer, seems to be related to signaling pathways such as the inhibition of the secretion of transforming growth factor β, inducing cellular apoptosis [29]. With this study, a strong possibility is that this cellular pathway is not as relevant in feline breast cancer. Thus, it is easy to understand that studies on other signaling pathways of feline breast cancer, which differs from humans, are needed. Nevertheless, 5-FU was effective in feline cells. Typically, this drug enters the cell and is intracellularly converted into fluorodeoxyuridine monophosphat, fluorodeoxyuridine triphosphate, and fluorouridine triphosphate. These metabolites inhibit thymidylate synthase and disrupt DNA and RNA synthesis [30,31]. In sum, in addition to all the aggressiveness underlying the FMCm cell line, molecular pathways different from those observed in human breast cancer need to be explored in more detail. Drug repurposing and combination, as observed in this study, are interesting techniques in this segment, providing new insights into possible molecular pathways present in this type of cancer, such as differences between breast cancer in humans and felines.

## 4. Conclusions

In Feline Mammary Carcinoma cells, our results demonstrated that no combination of drugs had any advantage over the respective individual drugs, contrasting with the human breast cancer MCF-7 cells. This may most likely be due to the characteristics of this cell line, which is particularly aggressive and highly tumorigenic and metastatic. Indeed, the obtained results with chloroquine were very revealing of the resistance of FMCm cells, contrasting with the high loss of cellular viability observed with this drug in MCF-7 cells. In fact, this drug was very active in MCF-7, in agreement with the literature [34], but inactive in FMCm cells, and for the first time this evidence was reported in this project. In sum, the feline neoplastic cells showed considerable resistance to the drugs and drug combinations tested in this study when compared with MCF-7 cells, suggesting that molecular pathways involved in breast cancer differ highly between feline and human cancers, being important to explore this difference in more detail. As observed in this study, drug repurposing and drug combination are interesting approaches to provide new insights about differences in feline and human breast cancer, opening doors to more detailed studies focusing on molecular pathways and pharmacodynamics.

## Figures and Tables

**Table 1:** Repurposed drugs used in this project, as well as their major clinical indication, pharmacodynamics, and mechanism of action [14]. Some examples of studies in breast cancer with these drugs are also presented.

Drug	Indication	Pharmacodynamics	Mechanism of Action	Examples of Evidence in Breast Cancer
Verapamil	Angina Cardiac arrhythmias Cardiomyopathies Hypertension	Blocks calcium channels.	Inhibits calcium channels of L-type by binding to a specific area of their alpha-1 subunit.	Verapamil is a P-Glycoprotein inhibitor and leads to enhanced sensitivity levels of breast cancer cells to other drugs, such as paclitaxel [15].
Aspirin	Pain Fever Inflammation Reduction of risk of myocardial infarction	Destabilizes the production of prostaglandins by targeting cyclooxygenase (COX)-1 and -2.	By binding COX enzymes, disrupts the conversion of arachidonic acid to thromboxane A2.	In MCF-7 cells, aspirin in combination with tamoxifen led to cell cycle arrest (in G0/G1 phase of cell cycle), decreasing the levels of cyclinD1 [16].
Losartan	Hypertension Reduction of the risk of stroke	Blocks Angiotensin II receptor.	Impedes angiotensin II binding to the receptor AT1 (Angiotensin Receptor Type I) in tissues like the adrenal gland.	By targeting AT1, this drug inhibits breast cancer cells proliferation and the progression of the cancer to an invasive phenotype [17].
Chloroquine	Infections of *P. vivax*, *P. malariae*, *P. ovale*, and *P. falciparum.* Off label for rheumatic diseases	Inhibits heme polymerase.	Inhibits heme polymerase, disrupting the conversion of heme to hemazoin, process that kills the parasite.	By blocking KCNH1 channels, this drug decreases migration of MDA-MB-231 breast cancer cells [18].
Cimetidine	Acid-reflux disorders Peptic ulcer disease Heartburn Acid indigestion	Antagonizes Histamine H2-receptor.	Binds to an H2-receptor in gastric cells, blocking histamine effects.	In breast cancer cells, Cimetidine can decrease cell adhesion, presenting antimetastatic effects [19].
Itraconazole	Fungal infections, such as aspergillosis and onychomycosis	Inhibits the fungal enzyme cytochrome P450 14α-demethylase.	Interacts with 14-α demethylase, necessary to convert lanosterol to ergosterol and maintain fungal viability.	Itraconazole inhibits the Hedgehog pathway, leading to elevated levels of cell death [20].
Isoniazid	Mycobacterial infections, such as *M. tuberculosis*	Inhibits the production of bacterial mycolic acids.	In, *M. tuberculosis*, inhibits InhA (enoyl reductase).	Few studies in breast cancer. However, being an inhibitor of monoamine oxidase A, it may improve outcomes in prostate cancer, where elevated levels of this enzyme correlate with aggressiveness [21].
Pravastatin	Hyperlipidemia, Prevention of some coronary events	Decreases the plasmatic concentration of low-density lipoprotein (LDL) cholesterol.	Inhibits HMG-CoA reductase, reducing cholesterol levels.	Statins promote cell cycle arrest and increased reactive oxygen species (ROS) production, enhancing oxidative stress [22].
Tacrine	Alzheimer’s Disease	Enhances cholinergic function.	Inhibits cholinesterase enzymes, leading to prolonged action of acetylcholine.	The conjugation of this drug with model amphipathic peptide (MAP) (leads to high toxicity levels to MCF-7 cells [23].

**Table 2:** IC_50_ values of 5-FU, verapamil, itraconazole and chloroquine in MCF-7 cells.

Drug	IC_50_ (μM)
5-FU	11.8 ± 1.8
Verapamil	29.5 ± 4.5
Itraconazole	2.1 ± 0.6
Chloroquine	16.0 ± 2.3

**Table 3:** Brief description of the P- and E-cadherin molecules.

Molecule	Description
P-cadherin	Cell-to-cell adhesion glycoprotein. Overexpression is connected to tumor-enhancing effects in breast cancer and other types of cancer, such as ovarian [32].
E-cadherin	Mediates cell adhesion and junction formations, being a part of the adherents junctions. In a general way, loss/decrease of its expression is linked with tumor progression and metastatic lesions [33].

## Data Availability

Not applicable.
